# Using Ambulatory Voice Monitoring to Investigate Common Voice Disorders: Research Update

**DOI:** 10.3389/fbioe.2015.00155

**Published:** 2015-10-16

**Authors:** Daryush D. Mehta, Jarrad H. Van Stan, Matías Zañartu, Marzyeh Ghassemi, John V. Guttag, Víctor M. Espinoza, Juan P. Cortés, Harold A. Cheyne, Robert E. Hillman

**Affiliations:** ^1^Center for Laryngeal Surgery and Voice Rehabilitation, Massachusetts General Hospital, Boston, MA, USA; ^2^Department of Surgery, Harvard Medical School, Boston, MA, USA; ^3^MGH Institute of Health Professions, Massachusetts General Hospital, Boston, MA, USA; ^4^Department of Electronic Engineering, Universidad Técnica Federico Santa María, Valparaíso, Chile; ^5^Computer Science and Artificial Intelligence Laboratory, Massachusetts Institute of Technology, Cambridge, MA, USA; ^6^Department of Music and Sonology, Faculty of Arts, Universidad de Chile, Santiago, Chile; ^7^Bioacoustics Research Laboratory, Laboratory of Ornithology, Cornell University, Ithaca, NY, USA

**Keywords:** voice monitoring, accelerometer, vocal function, voice disorders, vocal hyperfunction, glottal inverse filtering, machine learning

## Abstract

Many common voice disorders are chronic or recurring conditions that are likely to result from inefficient and/or abusive patterns of vocal behavior, referred to as vocal hyperfunction. The clinical management of hyperfunctional voice disorders would be greatly enhanced by the ability to monitor and quantify detrimental vocal behaviors during an individual’s activities of daily life. This paper provides an update on ongoing work that uses a miniature accelerometer on the neck surface below the larynx to collect a large set of ambulatory data on patients with hyperfunctional voice disorders (before and after treatment) and matched-control subjects. Three types of analysis approaches are being employed in an effort to identify the best set of measures for differentiating among hyperfunctional and normal patterns of vocal behavior: (1) ambulatory measures of voice use that include vocal dose and voice quality correlates, (2) aerodynamic measures based on glottal airflow estimates extracted from the accelerometer signal using subject-specific vocal system models, and (3) classification based on machine learning and pattern recognition approaches that have been used successfully in analyzing long-term recordings of other physiological signals. Preliminary results demonstrate the potential for ambulatory voice monitoring to improve the diagnosis and treatment of common hyperfunctional voice disorders.

## Introduction

Voice disorders have been estimated to affect approximately 30% of the adult population in the United States at some point in their lives, with 6.6–7.6% of individuals affected at any given point in time (Roy et al., 2005; Bhattacharyya, 2014). While many vocally healthy speakers take verbal communication for granted, individuals suffering from voice disorders experience significant communication disabilities with far-reaching social, professional, and personal consequences (NIDCD, 2012).

Normal voice sounds are produced in the larynx by rapid air pulses that are emitted as the vocal cords (folds) are driven into vibration by exhaled air from the lungs. Disturbances in voice production (i.e., voice disorders) can be caused by a variety of conditions that affect how the larynx functions to generate sound, including (1) neurological disorders of the central (Parkinson’s disease, stroke, etc.) or peripheral (e.g., damage to laryngeal nerves causing vocal fold paresis/paralysis) nervous system; (2) congenital (e.g., restrictions in normal development of laryngeal/airway structures) or acquired organic (laryngeal cancer, trauma, etc.) disorders of the larynx and/or airway; and (3) behavioral disorders involving vocal abuse/misuse that may or may not cause trauma to vocal fold tissue (e.g., nodules). The most frequently occurring subset of voice disorders is associated with *vocal hyperfunction*, which refers to chronic “conditions of abuse and/or misuse of the vocal mechanism due to excessive and/or ‘imbalanced’ [uncoordinated] muscular forces” (Hillman et al., 1989, p. 373). Over the years, our group has begun to provide evidence for the concept that there are two types of vocal hyperfunction that can be quantitatively described and differentiated from each other and normal voice production using a combination of acoustic and aerodynamic measures (Hillman et al., 1989, 1990).

*Phonotraumatic vocal hyperfunction* (previously termed adducted hyperfunction) is associated with the formation of benign vocal fold lesions – such as nodules and polyps. Vocal fold nodules or polyps are believed to develop as a reaction to persistent tissue inflammation, chronic cumulative vocal fold tissue damage, and/or environmental influences (Titze et al., 2003; Czerwonka et al., 2008; Karkos and McCormick, 2009). Once formed, these lesions may prevent adequate vocal fold contact/closure that reduces the efficiency of sound production and can cause individuals to compensate by increasing muscular and aerodynamic forces. This compensatory behavior may result in further tissue damage and become habitual due to the need to constantly maintain functional voice production during daily life in the presence of a vocal fold pathology. By contrast, *non-phonotraumatic vocal hyperfunction* (previously termed non-adducted hyperfunction) – often diagnosed as muscle tension dysphonia (MTD) or functional dysphonia – is associated with symptoms such as vocal fatigue, excessive intrinsic/extrinsic neck muscle tension and discomfort, and voice quality degradation in the absence of vocal fold tissue trauma. There can be a wide range of voice quality disturbances (e.g., various degrees of strain or breathiness) whose nature and severity can display significant situational variation, such as variation associated with changes in levels of emotional stress throughout the course of a day (Hillman et al., 1990). MTD can be triggered by a variety of conditions/circumstances, including psychological conditions (traumatizing events, emotional stress, etc.), chronic irritation of the laryngeal and/or pharyngeal mucosa (e.g., laryngopharyngeal reflux), and habituation of maladaptive behaviors, such as persistent dysphonia following resolution of an upper respiratory infection (Roy and Bless, 2000).

To assess the prevalence and persistence of hyperfunctional vocal behaviors during diagnosis and management, clinicians currently rely on patient self-report and self-monitoring, which are highly subjective and prone to be unreliable. In addition, investigators have studied clinician-administered perceptual ratings of voice quality and endoscopic imaging and the quantitative analysis of objective measures derived from acoustics, electroglottography, imaging, and aerodynamic voice signals (Roy et al., 2013). Among work that sought to automatically detect voice disorders, including vocal hyperfunction, acoustic analysis approaches have employed neural maps (Hadjitodorov et al., 2000), non-linear measures (Little et al., 2007), and voice source-related properties (Parsa and Jamieson, 2000) from snapshots of phonatory recordings obtained during a single laboratory session. Because hyperfunctional voice disorders are associated with daily behavior, the diagnosis and treatment of these disorders may be greatly enhanced by the ability to unobtrusively monitor and quantify vocal behaviors as individuals go about their normal daily activities. Ambulatory voice monitoring may enable clinicians to better assess the role of vocal behaviors in the development of voice disorders, precisely pinpoint the location and duration of abusive and/or maladaptive behaviors, and objectively assess patient compliance with the goals of voice therapy.

This paper reports on our ongoing investigation into the use of a miniature accelerometer on the neck surface below the larynx to acquire and analyze a large set of ambulatory data from patients with hyperfunctional voice disorders (before and after treatment stages) as compared to matched-control subjects. We have previously reported on our development of a user-friendly and flexible platform for voice health monitoring that employs a smartphone as the data acquisition platform connected to the accelerometer (Mehta et al., 2012b, 2013). The current report extends on that pilot work and describes data acquisition protocols, as well as initial results from three analysis approaches: (1) existing ambulatory measures of voice use, (2) aerodynamic measures based on glottal airflow estimates extracted from the accelerometer signal, and (3) classification based on machine learning and pattern recognition techniques. Although the methodologies of these analysis approaches largely have been published, the novel contributions of the current paper include ambulatory voice measures from the largest cohort of speakers to date (142 subjects), initial estimation of ambulatory glottal airflow properties, and updated machine learning results for the classification of 51 speakers with phonotraumatic vocal hyperfunction from matched-control speakers.

## Materials and Methods

This section describes subject recruitment, data acquisition protocols, and the three analysis approaches of existing voice use measures, aerodynamic parameter estimation, and machine learning to aid in the classification of hyperfunctional vocal behaviors.

### Subject Recruitment

Informed consent was obtained from all the subjects participating in this study, and all experimental protocols were approved by the institutional review board of Partners HealthCare System at Massachusetts General Hospital.

Two groups of individuals with voice disorders are being enrolled in the study: patients with phonotraumatic vocal hyperfunction (vocal fold nodules or polyps) and patients with non-phonotraumatic vocal hyperfunction (MTD). Diagnoses are based on a complete team evaluation by laryngologists and speech-language pathologists at the Massachusetts General Hospital Voice Center that includes (1) a complete case history, (2) endoscopic imaging of the larynx (Mehta and Hillman, 2012), (3) aerodynamic and acoustic assessment of vocal function (Roy et al., 2013), (4) patient-reported voice-related quality of life (V-RQOL) questionnaire (Hogikyan and Sethuraman, 1999), and (5) clinician-administered consensus auditory-perceptual evaluation of voice (CAPE-V) assessment (Kempster et al., 2009).

Matched-control groups are obtained for each of the two patient groups. Each patient typically aids in identifying a work colleague of the same gender and approximate age (±5 years) who has a normal voice. The normal vocal status of all control subjects is verified via interview and a laryngeal stroboscopic examination. Each control subject is monitored for one full 7-day week.

**Figure 1:**
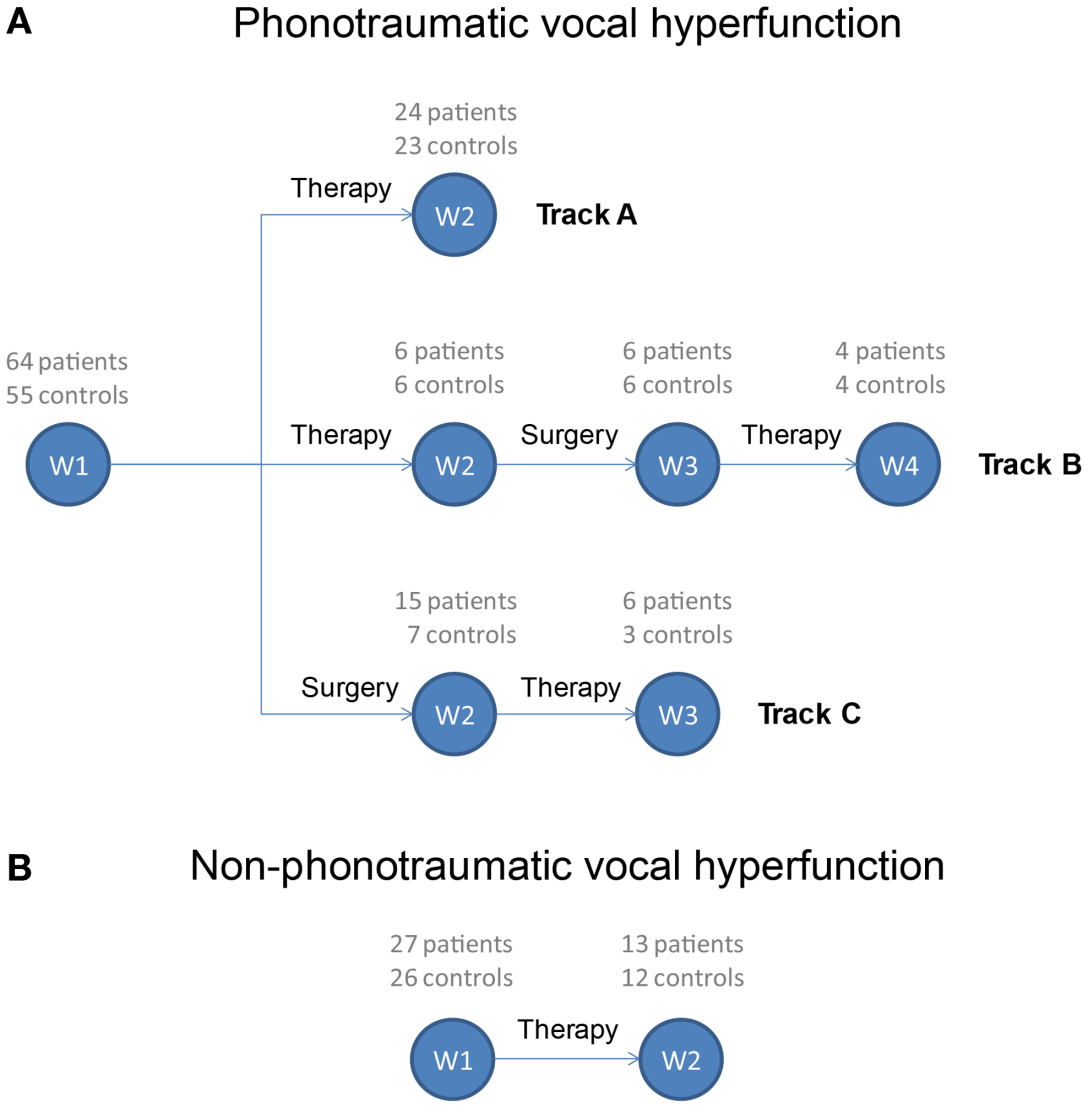
**Treatment tracks for patients exhibiting **(A)** phonotraumatic and **(B)** non-phonotraumatic hyperfunctional vocal behaviors**. Week numbers (W1, W2, W3, and W4) refer to time points during which ambulatory monitoring of voice use is being acquired using the smartphone-based voice health monitor. The current enrollment of each patient and matched-control pairing is listed above each week number.

Figure 1 displays the treatment sequences (tracks) and time points at which patients in the study are monitored for a full week. Patients with phonotraumatic vocal hyperfunction may follow one of three usual treatment tracks (Figure 1A). The particular treatment track chosen depends upon clinical management decisions regarding surgery or voice therapy. In Track A, individuals are monitored before and after successful voice therapy and do not need surgical intervention (therapy may involve sessions spanning several weeks or months). In Track B, patients initially attempt voice therapy but subsequently require surgical removal of their vocal fold lesion(s) to attain a more satisfactory vocal outcome; a second round of voice therapy is then typically required to retrain the vocal behavior of these patients to prevent the recurrence of vocal fold lesions. In Track C, patients undergo surgery first followed by voice therapy. Finally, patients with non-phonotraumatic vocal hyperfunction typically follow one treatment track and thus are monitored for 1 week before and after voice therapy (Figure 1B).

Data collection is ongoing, as Figure 1 lists patient enrollment along with the number of vocally healthy speakers who have been able to be recruited to be matched to a patient. For an initial analysis of a complete data set, results are presented for patients with available data from matched-control subjects. In addition, because the prevalence of these types of voice disorders is much higher in females (hence, more data acquired from female subjects) and to eliminate the impact on the analysis of known differences between male and female voice characteristics (such as fundamental frequency), only female subject data were of focus in the current report.

Table 1 lists the occupations and diagnoses of the 51 female participants with phonotraumatic vocal hyperfunction in the study who have been paired with matched-control subjects (there were only 4 male subject pairs). All participants were engaged in occupations considered to be at a higher-than-normal risk for developing a voice disorder. The majority of patients (37) were professional, amateur, or student singers; every effort was made to match singers with control subjects in a similar musical genre (classical or non-classical) to account for any genre-specific vocal behaviors. Forty-four patients were diagnosed with vocal fold nodules, and seven patients had a unilateral vocal fold polyp. The average (SD) age of participants within the group was 24.4 (9.1) years.

**Table 1:** **Occupations of adult females with phonotraumatic vocal hyperfunction and matched-control participants analyzed (51 pairs)**.

Occupation	No. of subject pairs	Patient diagnosis
Singer	37	Nodules (32) Polyp (5)
Teacher	5	Nodules
Consultant	2	Nodules (1) Polyp (1)
Psychotherapist/psychologist	2	Nodules
Recruiter	2	Nodules
Marketer	1	Nodules
Media relations	1	Nodules
Registered nurse	1	Polyp

*Diagnoses for the patient group are also listed for each occupation*.

Table 2 lists the occupations of the 20 female participants with non-phonotraumatic vocal hyperfunction in the study who have been paired with matched-control subjects (there were 6 male subject pairs). All patients were diagnosed with MTD and did not exhibit vocal fold tissue trauma. The average (SD) age of participants within the patient group was 41.8 (15.4) years.

**Table 2:** **Occupations of adult females with non-phonotraumatic vocal hyperfunction and matched-control participants analyzed (20 pairs)**.

Occupation	No. of subject pairs
Registered nurse	3
Singer	3
Teacher	3
Administrator	2
At-home caregiver	2
Student	2
Social worker	1
Actress	1
Administrative assistant	1
Exercise instructor	1
Systems analyst	1

*All patients were diagnosed with muscle tension dysphonia*.

### Data Acquisition Protocol

Prior to in-field ambulatory voice monitoring, subjects are assessed in the laboratory to document their vocal status and record signals that enable the calibration of the accelerometer signal for input to the vocal system model that is used to estimate aerodynamic parameters.

#### In-Laboratory Voice Assessment

**Figure 2:**
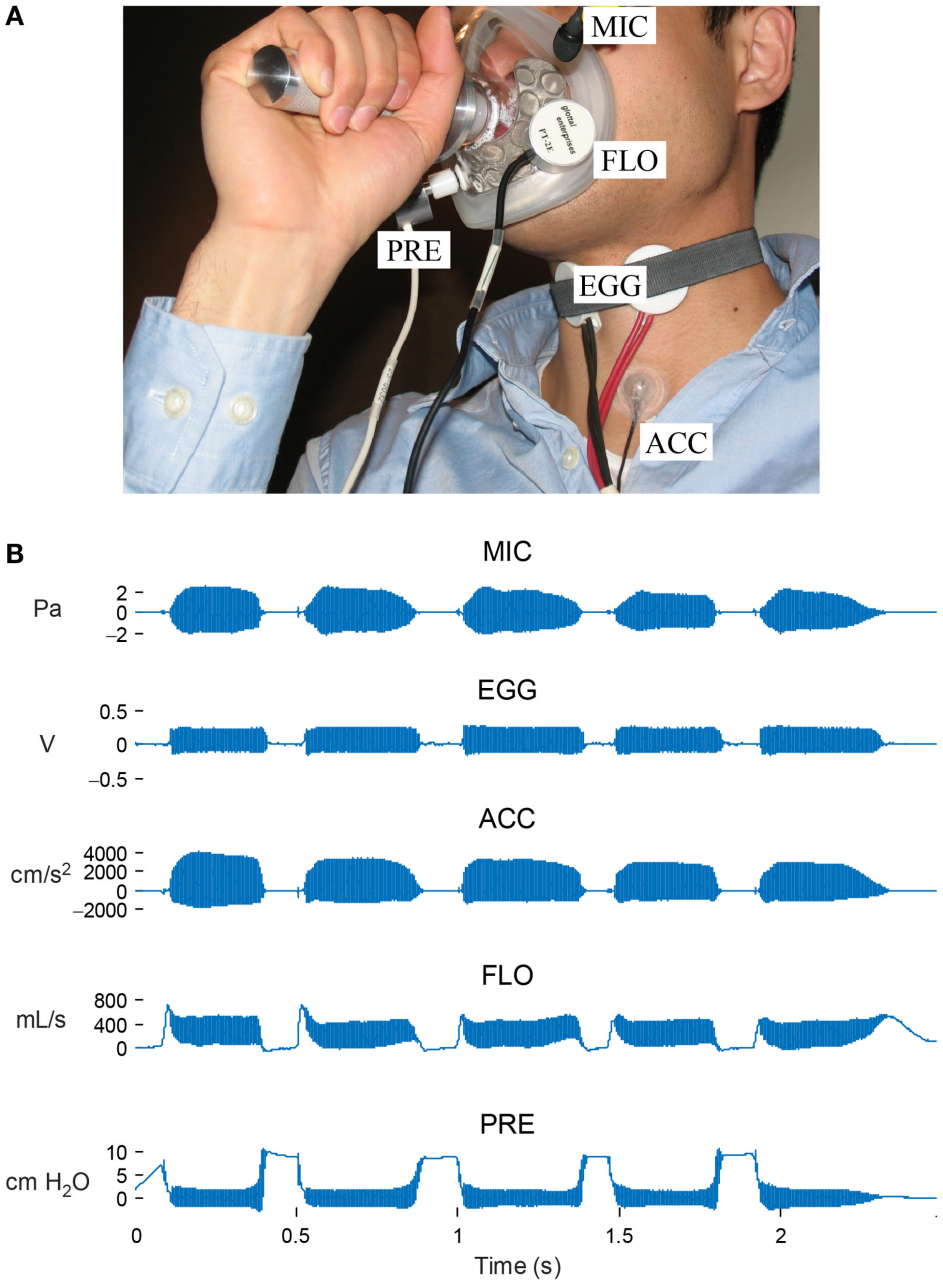
**In-laboratory data acquisition setup**. **(A)** Synchronized recordings are made of signals from an acoustic microphone (MIC), electroglottography electrodes (EGG), accelerometer sensor (ACC), high-bandwidth oral airflow (FLO), and intraoral pressure (PRE). **(B)** Signal snapshot of a string of “pae” tokens required for the estimation of subglottal pressure and airflow during phonation. © 2013 IEEE. Reprinted, with permission, from Mehta et al. (2013).

Figure 2A illustrates the in-laboratory multisensor setup consisting of the simultaneous acquisition of data from the following devices:

(1) Acoustic microphone placed 10 cm from the lips (MKE104, Sennheiser, Electronic GmbH, Wennebostel, Germany).
(2) Electroglottograph electrodes placed across the thyroid cartilage to measure time-varying laryngeal impedance (EG-2, Glottal Enterprises, Syracuse, NY, USA).
(3) Accelerometer placed on the neck surface at the base of the neck (BU-27135; Knowles Corp., Itasca, IL, USA).
(4) Airflow sensor collecting high-bandwidth aerodynamic data via a circumferentially vented pneumotachograph face mask (PT-2E, Glottal Enterprises).
(5) Low-bandwidth air pressure sensor connected to a narrow tube inserted through the lips in the mouth (PT-25, Glottal Enterprises).

In particular, the use of the pneumotachograph mask to acquire the high-bandwidth oral airflow signal is a key step in calibrating/adjusting the vocal system model described in Section “Estimating Aerodynamic Properties from the Accelerometer Signal” so that aerodynamic parameters can be extracted from the accelerometer signal (Zañartu et al., 2013). All subjects wore the accelerometer below the level of the larynx (subglottal) on the front of the neck just above the sternal notch. When recorded from this location, the accelerometer signal of an unknown phrase is unintelligible. The accelerometer sensor used is relatively immune to environmental sounds and produces a voice-related signal that is not filtered by the vocal tract, alleviating confidentiality concerns because speech audio is not recorded.

The in-laboratory protocol requires subjects to perform the following speech tasks at a comfortable pitch in their typical speaking voice mode:

(1) three cardinal vowels (“ah,” “ee,” “oo”) sustained at soft, comfortable, and loud levels;
(2) first paragraph of the Rainbow Passage at a comfortable loudness level;
(3) string of consonant-vowel pairs (e.g., “pae pae pae pae pae”).

The sustained vowels provide data for computing objective voice quality metrics such as perturbation measures, harmonics-to-noise ratio, and harmonic spectral tilt. The Rainbow Passage is a standard phonetically balanced text that has been frequently used in voice and speech research (Fairbanks, 1960). The string of /pae/ syllables is designed to enable non-invasive, indirect estimates of lung pressure (during lip closure for the /p/ when airway pressure reaches a steady state/equilibrates) and laryngeal airflow (during vowel production when the airway is not constricted) for a sustained vowel (Rothenberg, 1973). Figure 2B displays a snapshot of synchronized in-laboratory waveforms from the consonant-vowel task for a 28-year-old female music teacher diagnosed with vocal fold nodules.

#### In-Field Ambulatory Monitoring of Voice Use

In the field, an Android smartphone (Nexus S; Samsung, Seoul, South Korea) provides a user-friendly interface for voice monitoring, daily sensor calibration, and periodic collection of subject responses to queries about their vocal status (Mehta et al., 2012b). The smartphone contains a high-fidelity audio codec (WM8994; Wolfson Microelectronics, Edinburgh, Scotland, UK) that records the accelerometer signal using sigma-delta modulation (128× oversampling) at a sampling rate of 11 025 Hz. Of critical importance, operating system root access allows for control over audio settings related to highpass filtering and programmable gain arrays prior to analog-to-digital conversion. By default, highpass filter cutoff frequencies are typically set above 100 Hz to optimize cellphone audio quality and remove low-frequency noise due to wind noise and/or mechanical vibration. These cutoff frequencies undesirably affect frequencies of interest through spectral shaping and phase distortion; thus, for the current application, the highpass filter cutoff frequency is modified to a high-fidelity setting of 0.9 Hz. Smartphone rooting also enables setting the analog gain to maximize signal quantization; e.g., the WM8994 audio codec gain values can be set between −16.5 dB and +30.0 dB in increments of 1.5 dB.

**Figure 3:**
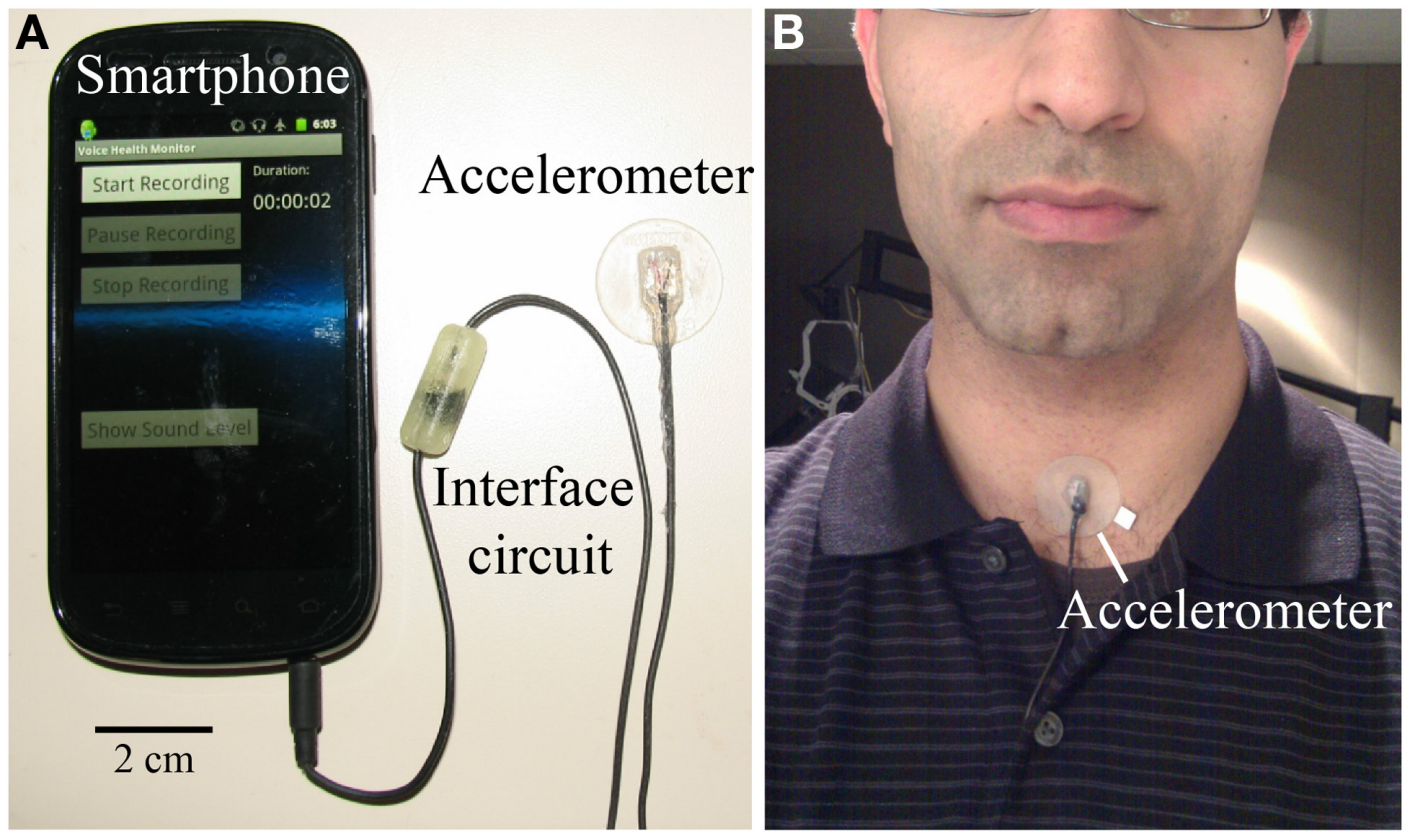
**Ambulatory voice health monitor: (A) smartphone, accelerometer sensor, and cable with interface circuit encased in epoxy; (B) the wired accelerometer mounted on a silicone pad affixed to the neck midway between the Adam’s apple and V-shaped notch of the collarbone**. © 2013 IEEE. Reprinted, with permission, from Mehta et al. (2013X).

Figure 3 displays the smartphone-based voice health monitor system. Each morning, subjects affix the accelerometer – encased in epoxy and mounted on a soft silicone pad – to their neck halfway between the thyroid prominence and the suprasternal notch using hypoallergenic double-sided tape (Model 2181, 3M, Maplewood, MN, USA). Smartphone prompts then lead the subject through a brief calibration sequence that maps the accelerometer signal amplitude to acoustic sound pressure level (Švec et al., 2005). Subjects produce three “ah” vowels from a soft to loud (or loud to soft) level that are used to generate a linear regression between acceleration amplitude and microphone signal level (decibel–decibel plot) so that the uncalibrated acceleration level can be converted to units of dB SPL (dB re 20 μPa). The acoustic signal is recorded using a handheld audio recorder (H1 Handy Recorder, Zoom Corporation, Tokyo, Japan) at a distance of 15 cm to the subject’s lips. The microphone is not needed the rest of the day.

With the smartphone placed in the pocket or worn in a belt holster, subjects engage in their typical daily activities at work and home and are able to pause data acquisition during activities that could damage the system, such as exercise, swimming, showering, etc. The smartphone application requires minimal user interaction during the day. Every 5 h, users are prompted to respond to three questions related to vocal effort, discomfort, and fatigue (Carroll et al., 2006):

(1) *Effort:* say “ahhh” softly at a pitch higher than normal. Then say “ha ha ha ha ha” in the same way. Rate how difficult the task was.
(2) *Discomfort:* what is your current level of discomfort when talking or singing?
(3) *Fatigue:* what is your current level of voice-related fatigue when talking or singing?

The three questions are answered using slider bars on the smartphone ranging from 0 (no presence of effort, discomfort, or fatigue) to 100 (maximum effort, discomfort, or fatigue).

At the end of the day, the accelerometer is removed, recording is stopped, and the smartphone is charged as the subject sleeps. A brief daily email survey asks subjects about when their work/school day began and ended and if anything atypical occurred during the day.

### Voice Quality and Vocal Dose Measures

Voice-related parameters for voice disorder classification fall into the following two categories: (1) time-varying trajectories of features that are computed on a frame-by-frame basis and (2) measures of voice use that accumulate frame-based metrics over a given duration (i.e., vocal dose measures). These measures may be computed offline in a *post hoc* analysis of data or online on the smartphone for real-time display or biofeedback.

**Figure 4:**
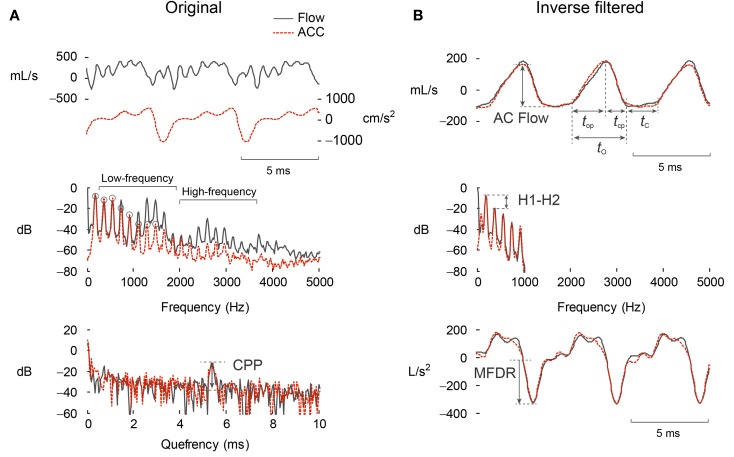
**Parameterization of the (A) original and (B) inverse-filtered waveforms from the oral airflow (black) and neck-surface acceleration (ACC, red-dashed) waveform processed with subglottal impedance-based inverse filtering**. Shown are the time waveform, frequency spectrum, and cepstrum, along with the parameterization of each domain to yield clinically salient measures of voice production.

Table 3 describes the suite of current frame-based parameters computed over 50-ms, non-overlapping frames. These modifiable frame settings currently mimic the default behavior of the Ambulatory Phonation Monitor (KayPENTAX, Montvale, NJ, USA) and strike a practical balance between the requirement of real-time computation and capture of temporal and spectral voice characteristics during time-varying speech production. The parameters quantify signal properties related to amplitude, frequency, periodicity, spectral tilt, and cepstral harmonicity: SPL and f0 (Mehta et al., 2012b), autocorrelation peak magnitude, harmonic spectral tilt (Mehta et al., 2011), low- to high-frequency spectral power ratio (LH ratio) (Awan et al., 2010), and cepstral peak prominence (CPP) (Mehta et al., 2012c). Figure 4A illustrates the computation of these measures from the time, spectral, and cepstral domains. In the past, we have set *a priori* thresholds on signal amplitude, fundamental frequency, and autocorrelation amplitudes to decide whether a frame contains voice activity or not (Mehta et al., 2012b). Since then, additional signal measures have been implemented to improve voice disorder classification and refine voice activity detection. Table 3 also reports the default ranges for each measure for a frame to be considered voiced.

**Table 3:** **Description of frame-based signal features computed on in-field ambulatory voice data**.

Feature	Units	Voicing criteria	Description
Sound pressure level at 15 cm	dB SPL	45–130	Acceleration amplitude mapped to acoustic sound pressure level (Švec et al., 2005)
Fundamental frequency	Hz	70–1000	Reciprocal of first non-zero peak location in the normalized autocorrelation function (Mehta et al., 2012b)
Autocorrelation peak amplitude		0.60–1	Relative amplitude of first non-zero peak in the normalized autocorrelation function (Mehta et al., 2012b)
Subharmonic peak		0.25–1	Relative amplitude of a secondary peak, if it exists, located around half way to the autocorrelation peak
Harmonic spectral tilt	dB/octave	−25–0	Linear regression slope over the first 8 spectral harmonics (Mehta et al., 2011)
Low-to-high spectral ratio	dB	22–50	Difference between spectral power below and above 2000 Hz (Awan et al., 2010)
Cepstral peak prominence	dB	10–35	Magnitude of the highest peak in the power cepstrum (Mehta et al., 2012c)
Zero crossing rate		0–1	Proportion of frame that signal crosses its mean

The development of accumulated vocal dose measures (Titze et al., 2003) was motivated by the desire to establish safety thresholds regarding exposure of vocal fold tissue to vibration during phonation, analogous to Occupational Safety and Health Administration guidelines for auditory noise and mechanical vibration exposure. The three most frequently used vocal dose measures to quantify accumulated daily voice use are phonation time, cycle dose, and distance dose. Phonation (voiced) time reflects the cumulative duration of vocal fold vibration, also expressed as a percentage of total monitoring time. The cycle dose is an estimate of the number of vocal fold oscillations during a given period of time. Finally, the distance dose estimates the total distance traveled by the vocal folds, combining cycle dose with vocal fold vibratory amplitude based on the estimates of acoustic sound pressure level.

Additionally, attempts were made to characterize vocal load and recovery time by tracking the occurrences and durations of contiguous voiced and non-voiced segments. From these data, occurrence and accumulation histograms provide a summary of voicing and silence characteristics over the course of a monitored period (Titze et al., 2007). To further quantify vocal loading, smoothing was performed over the binary vector of voicing decisions such that contiguous voiced segments were connected if they were close to each other based on a given duration threshold (typically <0.5 s). The derived contiguous segments approximate speech phrase segments produced on single breaths to begin to investigate respiratory factors in voice disorders (Sapienza and Stathopoulos, 1994).

Amplitude, frequency, and vocal dose features are traditionally believed to be associated with phonotraumatic hyperfunctional behaviors (e.g., talking loud, at an inappropriate pitch, or too much without enough voice rest) (Roy and Hendarto, 2005; Karkos and McCormick, 2009). However, our previous work demonstrated that overall average signal amplitude, fundamental frequency, and vocal dose measures were not different between 35 patients with vocal fold nodules or polyps and their matched-controls (Van Stan et al., 2015b). The results provided in this manuscript replicate our previous findings with a larger group of 51 matched pairs and extend the analysis approach by (1) adding novel measures related to voice quality and (2) completing novel comparisons among patients with non-phonotraumatic vocal hyperfunction versus matched controls and between both subtypes of patients with vocal hyperfunction.

### Estimating Aerodynamic Properties from the Accelerometer Signal

Subglottal impedance-based inverse filtering (IBIF) is a biologically inspired acoustic transmission line model that allows for the estimation of glottal airflow from neck-surface acceleration (Zañartu et al., 2013). This vocal system model follows a lumped-impedance parameter representation in the frequency domain using a series of concatenated *T*-equivalent segments of lumped acoustic elements that relate acoustic pressure to airflow. Each segment includes terms for representing key components for the subglottal system such as yielding walls (cartilage and soft tissue components), viscous losses, elasticity, and inertia. Then, a cascade connection is used to account for the acoustic transmission associated with the subglottal system based upon symmetric anatomical descriptions for an average male (Weibel, 1963). In addition, a radiation impedance is used to account for neck skin properties (Franke, 1951; Ishizaka et al., 1975) and accelerometer loading (Wodicka et al., 1989). The DC level of the airflow waveform is not modeled by IBIF due to the accelerometer waveform only being an AC signal. Thus, this overall approach provides an airflow-to-acceleration transfer function that is inverted when processing the accelerometer signal.

Subject-specific parameters need to be obtained to use subglottal IBIF as a signal processing approach for the accelerometer signal. Five parameters are estimated for each subject – three parameters for the skin model (skin inertance, resistance, and stiffness) and two parameters for tracheal geometry (tracheal length and accelerometer position relative to the glottis). The most relevant parameter values are searched for using an optimization scheme that minimizes the mean-squared error between oral airflow–derived and neck-surface acceleration–derived glottal airflow waveforms. A default parameter set is fine tuned to a given subject by means of five scaling factors *Q_i_* with *i* = 1, … , 5, which are designed to be estimated from a stable vowel segment. Since the subglottal system is assumed to remain the same for all other conditions (loudness, vowels, etc.), the estimated *Q* parameters may only need to be obtained once for each subject.

The subglottal IBIF scheme was initially evaluated for controlled scenarios that represented different glottal configurations and voice qualities in sustained vowel contexts (Zañartu et al., 2013). Under these conditions, a mean absolute error of <10% was observed for two glottal airflow measures of interest: maximum flow declination rate (MFDR) and the peak-to-peak glottal flow (AC Flow). Recently, the method was adapted for a real-time implementation in the context of ambulatory biofeedback (Llico et al., 2015), but again tested and validated only in sustained vowel contexts. Therefore, an evaluation of the subglottal IBIF method under continuous speech conditions is a natural next step. Continuous speech is the scenario where subglottal IBIF has the most potential to contribute to the field of voice assessment, as it can provide aerodynamic measures in the context of an ambulatory assessment of vocal function.

In this paper, we provide an initial assessment of the performance of the subglottal IBIF scheme for the phonetically balanced Rainbow Passage obtained in the laboratory, as well as for the data obtained from a weeklong recording in the field. Multiple measures of vocal function were extracted from each cycle and averaged over 50-ms frames (50% overlap), including AC Flow, MFDR, open quotient (OQ), speed quotient (SQ), spectral slope (H1–H2), and normalized amplitude quotient (NAQ). Figure 4 illustrates the extraction of these measures from the inverse-filtered acceleration waveform in the time and spectral domains. OQ is defined as *t*_O_/(*t*_O_ + *t*_C_), and SQ is defined as 100(*t*_op_/*t*_cp_). NAQ is a measure of the closing phase and is defined as the ratio of AC Flow to MFDR normalized by the period duration (*t*_O_ + *t*_C_) (Alku et al., 2002).

The in-laboratory voice assessment described in Section “In-Laboratory Voice Assessment” enables a direct comparison of the subglottal IBIF of neck-surface acceleration with vocal tract inverse-filtering of the oral airflow waveform. It is noted that inverse filtering of oral airflow for time-varying, continuous speech segments is a topic of research unto itself, and there are no clear guidelines to best approach the problem. Thus, we selected a simple but clinically relevant method of oral airflow processing based on single formant inverse filtering (Perkell et al., 1991) that has been used for the assessment of vocal function in speakers with and without voice disorders (Hillman et al., 1989; Perkell et al., 1994; Holmberg et al., 1995). Subglottal IBIF with a single set of *Q* parameters was used to estimate a continuous glottal airflow signal for each speaker’s ambulatory time series.

### Machine Learning and Pattern Recognition Approaches

Machine learning and pattern recognition approaches have become strong tools in the analysis of time series data. This has been particularly true in wireless health monitoring (Clifford and Clifton, 2012), where multiple levels of analysis are needed to abstract a clinically relevant diagnosis or state. Learning problems can be mapped onto a set of four general components: (1) choice of training data and evaluation method, (2) representation of examples (often called feature engineering), (3) choice of objective function and constraints, and (4) choice of optimization method. Choosing these components should be dictated by the goal at hand and the type of data available.

We first considered the case of patients with phonotraumatic vocal hyperfunction prior to any treatment and their matched controls. Each subject (patient or control) had a week of ambulatory neck-surface acceleration data related to voice use. Previous work suggested that long-term averages of standard voice measures did not capture differences between patients with vocal fold nodules or polyps and their matched controls (Mehta et al., 2012a). Thus we hypothesize that the tissue pathology (nodules or polyps) could create aggregate differences at the extremes of the recorded time series rather than at the averages. We had some initial success examining whether statistical features of fundamental frequency (f0) and SPL, such as skewness, kurtosis, 5th percentile, and 95th percentile, could capture this more extreme information and lead to an accurate patient classifier in our population.

Briefly, we first extracted SPL and f0 measures and voicing criteria described in Section “Voice Quality and Vocal Dose Measures” from 50-ms, non-overlapping frames. From these frames, we built 5-min, non-overlapping windows (i.e., 6000 frames per window) over each day in a subject’s entire weeklong record. We then took univariate statistics of feature histograms and the cumulative vocal dose measures from windows containing at least 30 frames labeled as voiced (0.5% phonation time). Normalized versions of the statistics were obtained by converting each statistic into units of SD based on that feature’s baseline distribution over an average hour in the first half of the day. Additional methodological details are available in a previous publication (Ghassemi et al., 2014).

Here, a concatenated feature matrix represented each subject’s week. The features from each 5-min window were associated with a patient or control label and used to create an L1-regularized logistic regression using a least absolute shrinkage and selection operator (LASSO) model. The LASSO model was used to classify 5-min windows from a held-out set of data from patient and control subjects. We used leave-one-out-cross-validation (LOOCV) to partition our dataset of 51 paired adult female subjects into 51 training and test sets such that a single patient-control pair was the held-out test set at each of the 51 iterations. If more than a given proportion of the test subject’s windows were classified with a patient label, we predicted that subject as being a patient; otherwise, the subject was classified as a normal control. Classification performance was evaluated across the 51 LASSO models by the proportion of the test set correctly predicted, as well as by the area under the receiver operating characteristic curve (AUC), *F*-score, sensitivity (correct labeling of patients), and specificity (correct labeling of controls).

## Results

Selected results from applying the three analysis approaches to the current data set of phonotraumatic and non-phonotraumatic vocal hyperfunction groups are reported as an initial demonstration of the potential discriminative performance and predictive power of these methods. Patients and their matched-control subjects continue to be enrolled and followed throughout their treatment stages.

### Summary Statistics of Voice Quality and Vocal Dose Measures

**Figure 5:**
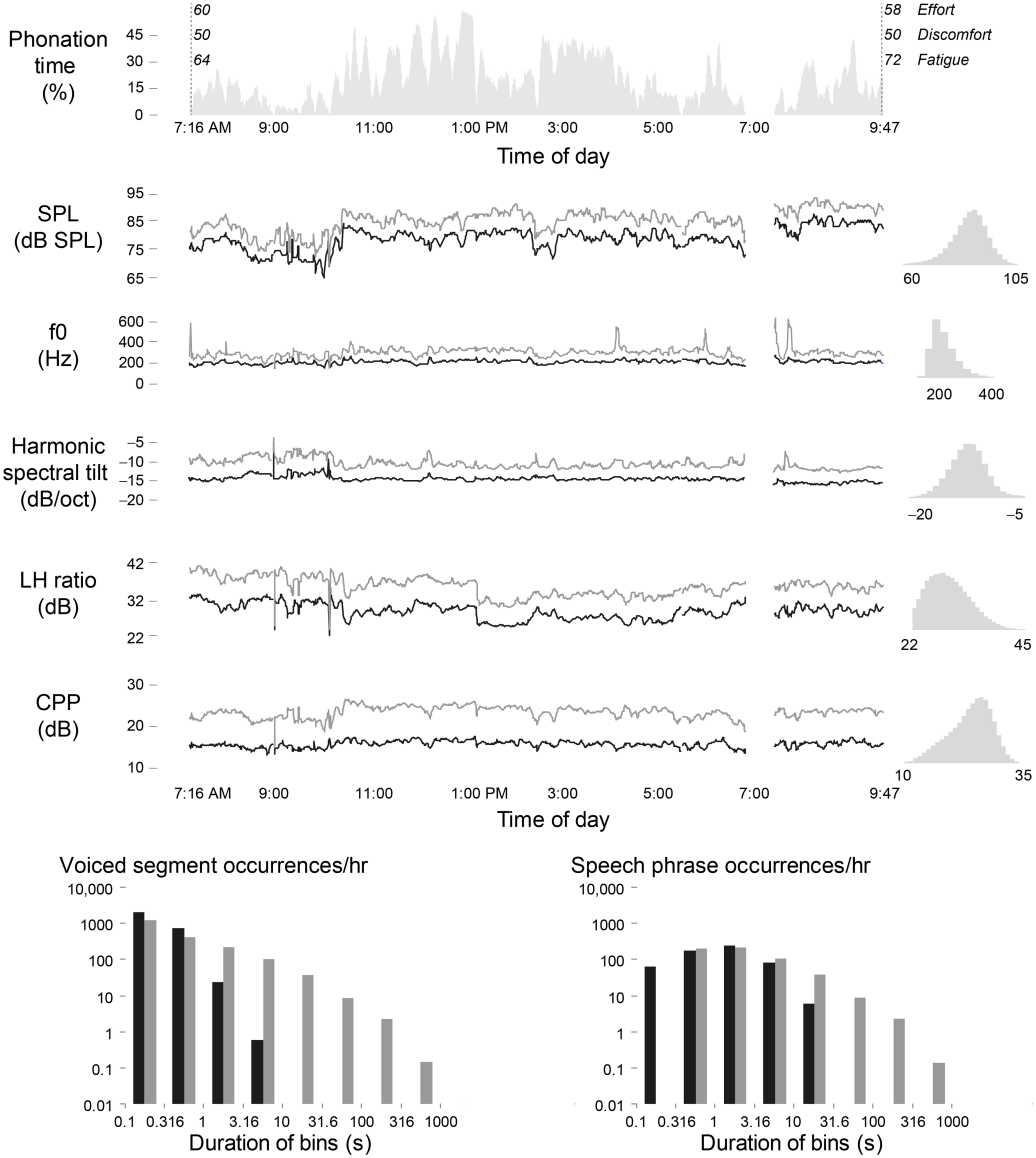
**Illustration of a daily voice use profile for an adult female diagnosed with bilateral vocal fold nodules**. Shown are 5-min moving averages of the median and 95th percentile of frame-based voice quality measures, along with self-reported ratings of effort, discomfort, and fatigue at the beginning and end of day. The daylong histograms of each measure are shown to the right of each time series. The plots below display the occurrence histograms of contiguous voiced segments (left) and estimates of speech phrases between breaths (right).

Figure 5 illustrates a daylong voice use profile of a 34-year-old adult female psychologist prior to surgery for a left vocal fold polyp and right vocal fold reactive nodule. Phonation time for her day reached 20.3% with a mean (SD) SPL of 81.8 (6.4) dB SPL and f0 mode (SD) of 194.5 (51.2) Hz. Such visualizations (made interactive through navigable graphical user interfaces) of measures such those described in Section “Voice Quality and Vocal Dose Measures” may ultimately enable clinicians to identify certain patterns of voice features related to vocal hyperfunction and subsequently make informed decisions regarding patient management.

As an initial description of the pre-treatment patient data, summary statistics were computed from the weeklong time series of SPL, f0, voice quality features, and vocal dose measures. The 5th percentiles and 95th percentiles were used to compute minimum, maximum, and range statistics. A four-factor, one-way analysis of variance was carried out for each summary statistic in the comparison of the two patient groups and their respective matched-control groups. The between-group comparisons consisted of the phonotraumatic patients versus their matched controls (51 pairs), the non-phonotraumatic patients versus their matched controls (20 pairs), and the phonotraumatic group versus the non-phonotraumatic group.

Table 4 reports the group-based mean (SD) for voice use summary statistics of SPL, f0, and vocal dose measures for weeklong data collected from the phonotraumatic patient and matched-control groups and the non-phonotraumatic patient and matched-control groups. Based on a *post hoc* analysis, measures that exhibited statistically significant differences (*p* < 0.001) between the two patient groups and between patient and matched-control groups are highlighted. The table also reports voice quality summary statistics of the autocorrelation peak magnitude, harmonic spectral tilt, LH ratio, and CPP.

**Table 4:** **Group-based mean (SD) of summary statistics of weeklong vocal dose and voice quality measures computed for adult females in the phonotraumatic (*n* = 51) and non-phonotraumatic (*n* = 20) vocal hyperfunction patient groups and their respective control groups**.

Summary statistic	Phonotraumatic controls	Phonotraumatic group	Non-phonotraumatic group	Non-phonotraumatic controls
Monitoring duration (hh:mm:ss)	81:11:49 (13:13:35)	77:21:43 (15:36:33)	73:44:37 (10:04:12)	78:59:16 (13:50:13)
**SPL (dB SPL re 15 cm)**				
Mean	83.9 (4.6)	85.2 (4.1)	80.1 (6.0)	83.0 (5.2)
SD	12.5 (2.4)	11.8 (1.9)	9.9 (3.1)	11.2 (3.3)
Minimum	62.7 (5.8)	64.5 (4.9)	63.3 (7.0)	64.5 (6.3)
Maximum	104.2 (6.7)	103.5 (5.9)	96.3 (8.3)	101.7 (9.5)
Range	41.4 (8.5)	39.0 (6.7)	33.0 (10.6)	37.2 (11.6)
**f0 (Hz)**				
Mode	201.4 (19.1)	197.2 (22.3)	193.8 (31.1)	192.9 (25.7)
SD	89.6 (17.5)	75.3 (17.3)	73.5 (24.9)	70.1 (14.3)
Minimum	170.3 (14.9)	166.7 (17.4)	160.0 (20.5)	163.2 (22.2)
Maximum	440.6 (58.9)	392.4 (65.5)	382.4 (81.4)	374.6 (62.3)
Range	270.3 (55.9)	225.7 (56.7)	222.4 (81.2)	211.4 (49.4)
**Phonation time**				
Cumulative (hh:mm:ss)	7:24:08 (2:33:32)	7:33:45 (2:36:34)	4:25:14 (2:31:57)	5:46:13 (2:16:17)
Normalized (%)	9.2 (2.9)	9.7 (2.6)	6.0 (3.1)	7.3 (2.7)
**Cycle dose**				
Cumulative (millions of cycles)	7.121 (2.76)	6.718 (2.495)	3.708 (2.202)	4.814 (1.831)
Normalized (cycles/h)	87,954 (30,508)	85,719 (25,633)	49,892 (26,997)	61,310 (22,241)
**Distance dose**				
Cumulative (m)	26,769 (11,815)	26,689 (10,999)	12,254 (8284)	18,084 (8466)
Normalized (m/h)	330.0 (129.3)	340.7 (112.1)	165.1 (102.4)	228.0 (98.4)
**Autocorrelation peak**				
Mean	0.851 (0.018)	0.843 (0.015)	0.827 (0.022)	0.837 (0.014)
SD	0.080 (0.004)	0.079 (0.004)	0.082 (0.007)	0.079 (0.004)
Minimum	0.677 (0.020)	0.672 (0.016)	0.657 (0.024)	0.668 (0.014)
Maximum	0.941 (0.010)	0.934 (0.011)	0.926 (0.014)	0.928 (0.010)
Range	0.263 (0.015)	0.262 (0.014)	0.269 (0.021)	0.260 (0.013)
**Harmonic spectral tilt (dB/oct)**				
Mean	−14.1 (0.6)	−14.4 (0.6)	−13.6 (1.1)	−14.1 (0.8)
SD	2.4 (0.3)	2.4 (0.2)	2.5 (0.3)	2.4 (0.2)
Minimum	−17.8 (0.8)	−18.2 (0.8)	−17.5 (1.0)	−17.8 (1.1)
Maximum	−9.9 (0.8)	−10.5 (0.6)	−9.3 (1.5)	−9.8 (1.0)
Range	8.0 (1.0)	7.7 (0.8)	8.2 (1.2)	8.0 (0.8)
**LH ratio (dB)**				
Mean	30.5 (1.1)	30.5 (1.3)	30.1 (1.3)	30.7 (1.5)
SD	4.4 (0.4)	4.5 (0.4)	4.1 (0.5)	4.5 (0.5)
Minimum	24.0 (0.6)	23.8 (0.7)	23.8 (0.5)	24.1 (0.7)
Maximum	38.3 (1.6)	38.6 (1.8)	37.3 (2.1)	38.8 (2.2)
Range	14.3 (1.3)	14.8 (1.3)	13.5 (1.7)	14.7 (1.6)
**CPP (dB)**				
Mean	22.9 (1.0)	23.2 (1.1)	21.4 (2.1)	22.8 (1.1)
SD	4.5 (0.3)	4.4 (0.3)	4.2 (0.5)	4.4 (0.3)
Minimum	15.1 (0.5)	15.3 (0.6)	14.3 (0.8)	14.9 (0.7)
Maximum	29.6 (1.2)	29.7 (1.2)	28.0 (2.3)	29.3 (1.1)
Range	14.5 (1.0)	14.4 (0.9)	13.8 (1.6)	14.4 (1.0)

*Statistically significant differences between means are highlighted (*p* < 0.001). Minimum, maximum, and range are trimmed estimators reporting 5th percentile, 95th percentile, and range of the middle 90% of the data, respectively*.

Individuals with vocal fold nodules or polyps exhibited statistically significant differences compared to individuals with MTD for all parameters except f0. Of note, except for a few instances, the patient groups and their respective matched-control groups had remarkably similar accumulated/averaged measurement values (i.e., few statistically significant differences). These results replicate previously reported findings that, on average, individuals with nodules or polyps do not speak more often, at a different vocal intensity, or at a different habitual pitch compared to matched individuals with healthy voices (Van Stan et al., 2015b). Furthermore, the results provide initial evidence that patients with MTD also do not differ in these metrics compared to their matched controls (although CPP trended toward being higher in the normative group). More sensitive approaches are thus warranted to increase the discriminatory power among the groups, and the applications of the next two analysis frameworks yield promising, complementary perspectives.

### Examples of Subglottal Impedance-Based Inverse Filtering

**Figure 6:**
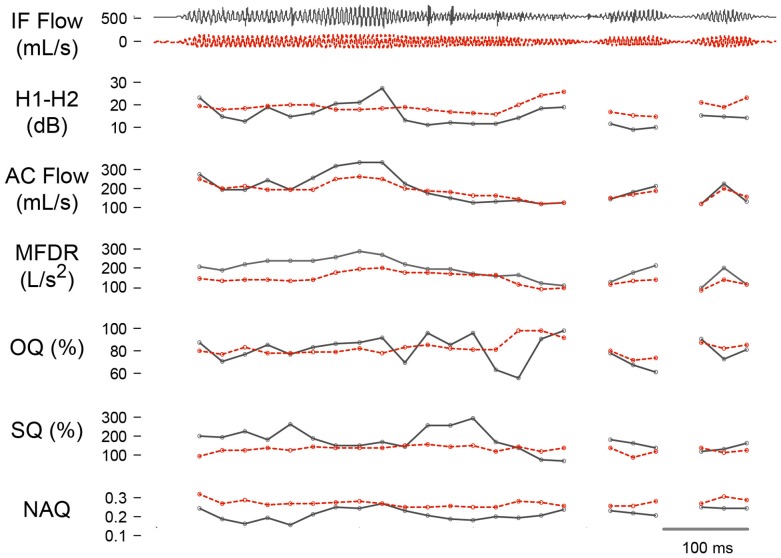
**Time-varying estimation of measures derived from the airflow-derived (black) and accelerometer-derived (red-dashed) glottal airflow signal using subglottal impedance-based inverse filtering**. Trajectories are shown for an adult female with no vocal pathology for the difference between the first two harmonic amplitudes (H1-H2), peak-to-peak flow (AC Flow), maximum flow declination rate (MFDR), open quotient (OQ), speed quotient (SQ), and normalized amplitude quotient (NAQ).

The results of both in-laboratory and in-field assessments are illustrated for a single normal female subject. The subglottal IBIF yielded estimates of glottal airflow from the neck-surface accelerometer for both assessments. Figure 6 shows a direct contrast of the glottal airflow estimates from oral airflow and neck-surface acceleration for a portion of the Rainbow Passage. Both waveforms and derived measures are presented, where it can be seen that, although the fit between signals can be adequate, the IBIF-based signal is less prone to inverse filtering artifacts than its oral airflow-based counterpart. This is due to the more stationary underlying dynamic behavior of the subglottal system relative to that of the time-varying vocal tract, thus constituting a more tractable inverse filtering problem. As a result, the measures of vocal function derived from the subglottal IBIF processing appear to be more reliable. Improving upon methods for inverse filtering of oral airflow in running speech is a current focus of research, which would also allow for testing the assumption that *Q* parameters in the IBIF scheme should remain constant in continuous speech conditions.

**Figure 7:**
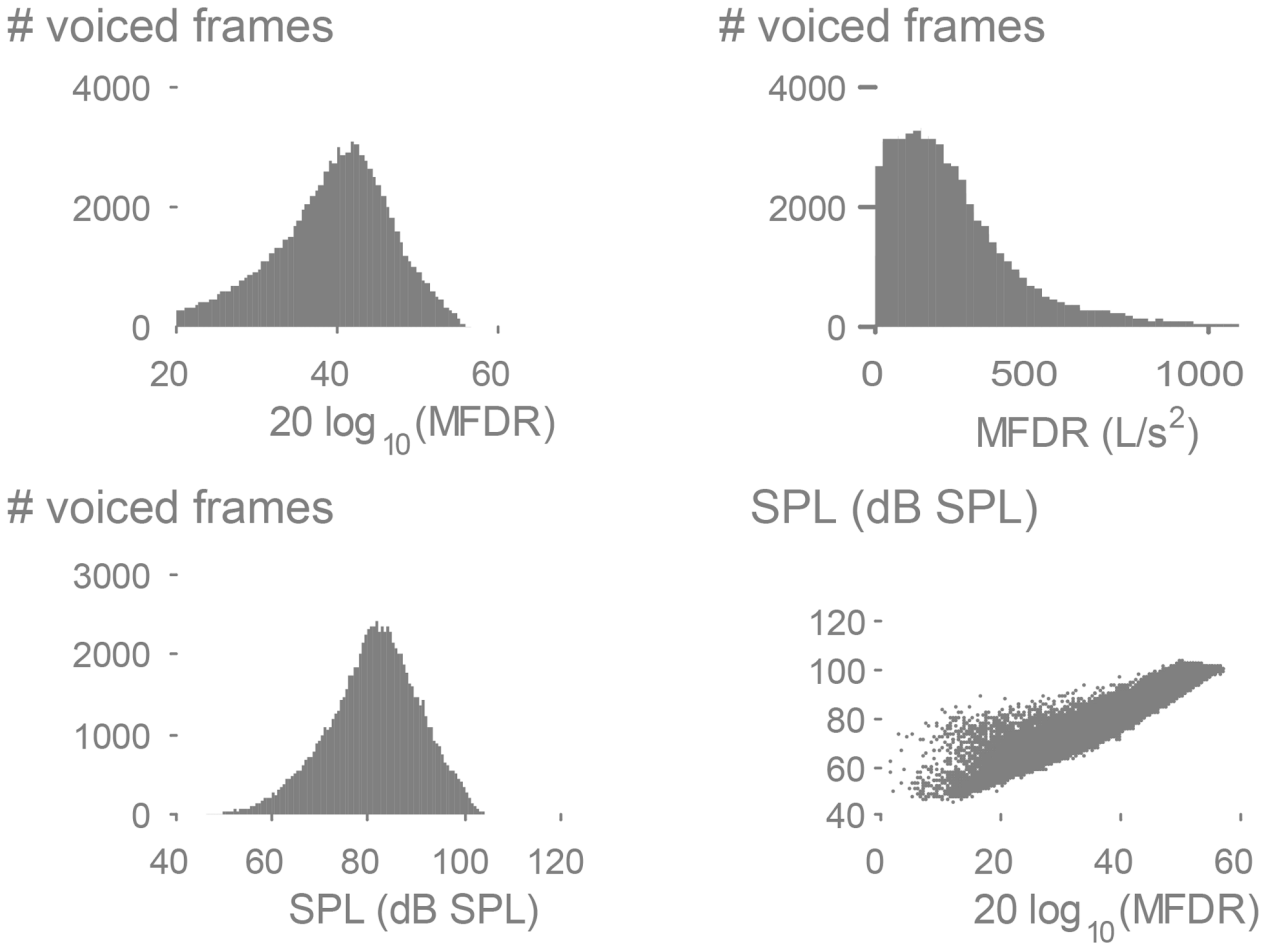
**Exemplary results using subglottal impedance-based inverse filtering of a weeklong neck-surface acceleration signal from an adult female with a normal voice**. Histograms of the maximum flow declination rate (MFDR) measure are displayed in physical and logarithmic units. The logarithm of MFDR is plotted against sound pressure level (SPL) to confirm the expected linear correlation (*r* = 0.94) and slope (1.13 dB/dB).

Figure 7 presents histograms of SPL and MFDR derived from the weeklong neck-surface acceleration recording. The SPL/MFDR relation provides insights on the efficiency in voice production, which was found to be 9 dB per MFDR doubling in sustained vowels for normal female subjects (6 dB per MFDR doubling for male subjects) (Holmberg et al., 1988). It is noted in Figure 7 that when a linear scale is used for MFDR, the histogram peak appears skewed to the left. However, when applying a logarithmic transform to MFDR (Holmberg et al., 1988, 1995), both SPL and MFDR histograms become Gaussian with different means and variances. The ambulatory relation provides a slope of 1.13 dB/dB, which is similar to the 1.5 dB/dB slope (9 dB per MFDR doubling) reported for oral airflow-based inverse filtering features under sustained vowel conditions (Holmberg et al., 1988). This result is encouraging as it provides initial validation for ambulatory MFDR estimation using subglottal IBIF and also provides an indication that average behaviors in normal subjects could be related to simple sustained vowel tasks in a clinical assessment. The relationship warrants further investigation, with challenges foreseen for subjects with voice disorders.

### Classification Results Using Machine Learning

**Figure 8:**
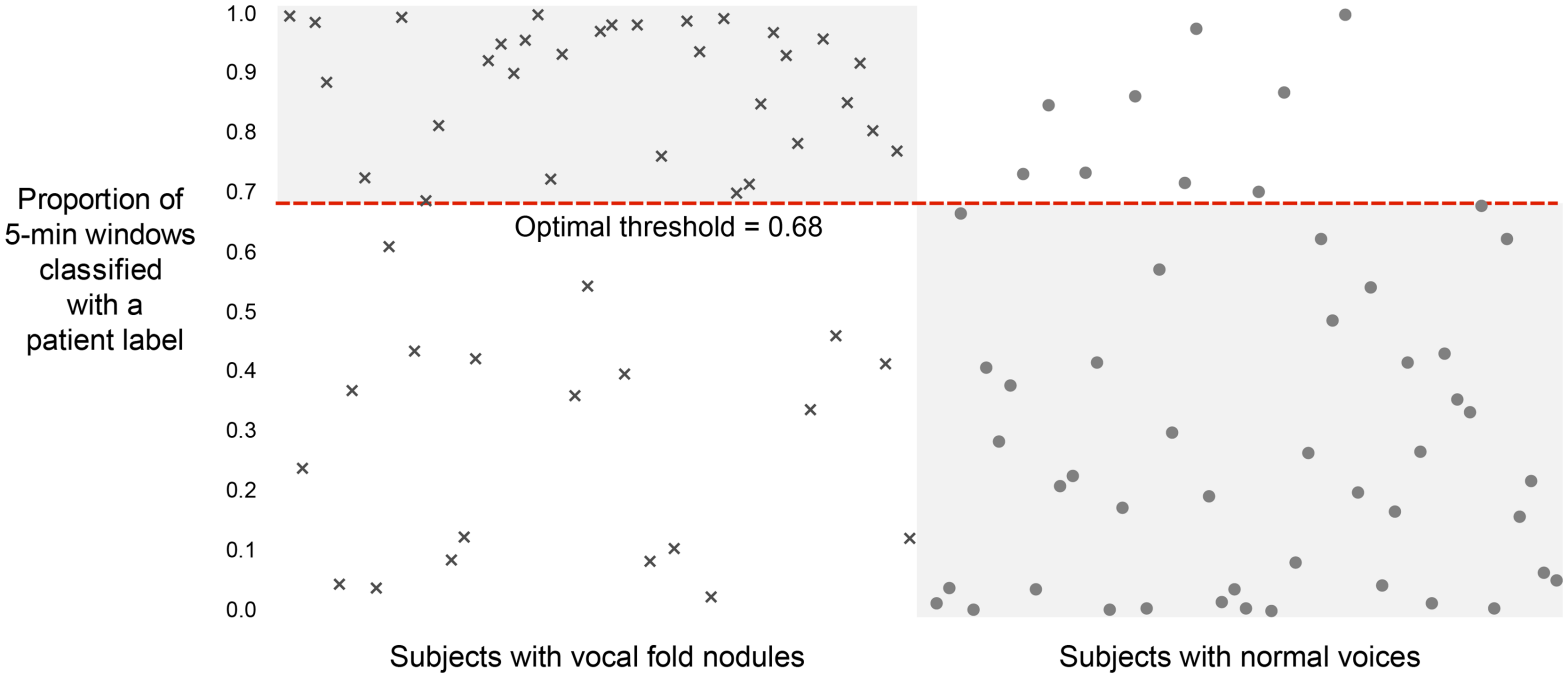
**Classification results on 102 adult female subjects, 51 with vocal fold nodules or polyps and 51 matched-control subjects with normal voices**. Per-patient unbiased model performance using summary statistics of sound pressure level and fundamental frequency from non-overlapping, 5-min windows.

Figure 8 shows that we were able to correctly classify 74 out of 102 subjects (72.5%) using a threshold of 0.68. Intuitively, this means that a subject is predicted to be a patient with phonotraumatic vocal hyperfunction if more than 68% of their windows were classified similarly to those from the other patients the LASSO model was trained on. The mean (SD) of performance across the 51 LASSO models was 0.739 (0.274) for AUC, 0.766 (0.204) for *F*-score, 0.739 (0.296) for sensitivity, and 0.767 (0.288) for specificity.

Table 5 summarizes the performance of the statistical measures in classifying phonotraumatic vocal hyperfunction. As shown, subjects with phonotrauma tended to have f0 and SPL distributions that were right-shifted from their previous values, i.e., an increased Normalized F0 95th percentile and an increased Normalized SPL Skew. We contrast this with the vocally normal group, which had a right-shifted (non-normalized) SPL distribution, i.e., increased SPL Skew. We could interpret the right-shifting of Normalized features in subjects with vocal fold nodules to mean that they tended to deviate from their baseline f0 and SPL as their days progressed, possibly reflecting increased difficulty in producing phonation. For the controls, the fact that their absolute SPL Skew was increased without a corresponding increase to their Normalized distribution suggests that even when control subjects exhibited higher SPL ranges, they tended to stay within their baseline ranges.

**Table 5:** **Association of summary statistics features of sound pressure level (SPL) and fundamental frequency (f0) with group label across the 51 LASSO models**.

	Association count		Multivariate LASSO association	
	
Summary statistic	Patient	Control	Beta mean (SD)	Odds ratio mean (95% CI)
Normalized SPL skew	51	0	1.11 (0.04)	3.03 (2.72–3.69)
Normalized f0 95th percentile	51	0	0.86 (0.03)	2.36 (2.16–2.70)
f0 skew	51	0	0.53 (0.09)	1.69 (1.42–2.35)
Normalized SPL kurtosis	51	0	0.28 (0.02)	1.32 (1.22–1.44)
Normalized SPL 5th percentile	51	0	0.14 (0.03)	1.16 (1.05–1.30)
Normalized percent phonation	51	0	0.12 (0.02)	1.13 (1.07–1.20)
Normalized f0 5th percentile	0	50	−0.10 (0.02)	0.91 (0.85–1.00)
Normalized SPL 95th percentile	0	51	−0.17 (0.03)	0.84 (0.77–0.91)
SPL kurtosis	0	51	−0.28 (0.02)	0.76 (0.69–0.82)
Normalized f0 skew	0	51	−0.41 (0.07)	0.66 (0.51–0.77)
SPL skew	0	51	−2.84 (0.12)	0.06 (0.03–0.08)

*The maximum number that the “association count” field can have is 51. This occurs when that particular variable (row) has a statistically significant effect (*p* < 0.001, absolute average odds ratios ≥1.10) in each model. Many associations persisted across all models and also tended to agree well on the magnitude of the association. The 95% confidence interval (CI) is from the lowest bound across subsets to the highest bound across subsets*.

While a majority of subjects were correctly classified in this framework, the predicted labels for some subjects are notably incorrect. One possible reason the classification is more accurate for the patient versus the control group (19 incorrectly labeled patients versus 9 incorrectly labeled controls) might stem from our strong labeling assumptions. It is likely that not all frames (and therefore not all statistical features of 5-min windows) of a patient exhibit vocal behavior associated with phonotraumatic hyperfunction. This creates a potentially large set of false-positive labels that can cause classification bias.

## Discussion

**Figure 9:**
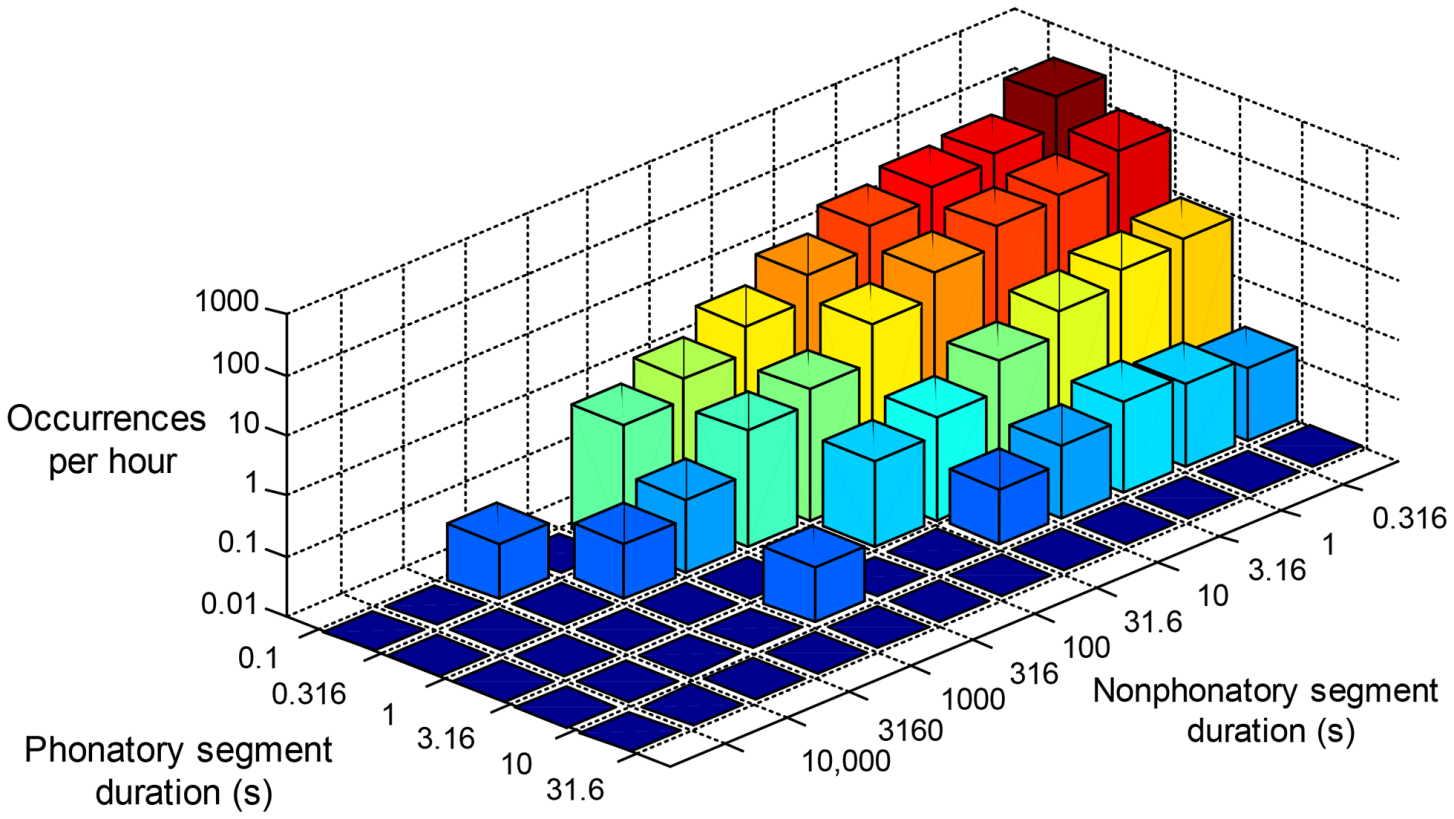
**Occurrence histogram of voiced/unvoiced contiguous segment pairs**. The figure includes the number of times (per hour) that a voiced segment of a given duration is followed by an unvoiced segment of a given duration.

An understanding of daily behavior is essential to improving the diagnosis and treatment of hyperfunctional voice disorders. Our results indicate that supervised machine learning techniques have the potential to be used to discriminate patients from control subjects with normal voice. It is important to note, however, that this work did not account for time of day, sequence of window occurrence, or ordered loading of features. For an example of time-ordered analysis, Figure 9 shows a three-dimensional distribution showing the occurrence histograms of unvoiced segment durations that immediately followed successively longer voiced-segment durations over the course of a day. This analysis approach attempts to reflect a speaker’s vocal behavior in terms of how much voice rest follows bursts of voicing activity. Similarly, ongoing monitoring of phonation time after a particular vocal load in a preceding window represents additional methods that may lead to complementary pieces of information that can aid in the successful detection of hyperfunctional vocal behaviors.

The subglottal IBIF measures for continuous speech appear more accurate than the oral airflow based due to the additional challenges associated with performing time-varying inverse filtering for the vocal tract. Improving upon methods for inverse filtering of oral airflow in continuous speech is a current focus of research, which would also allow for testing the assumption that *Q* parameters remain constant during speech production. The evaluation of subglottal IBIF using weeklong ambulatory data acquired with the VHM illustrates that the relation between SPL and MFDR is very well aligned with previous observations for sustained vowels for adult female subjects (Holmberg et al., 1988). This result provides initial validation of using IBIF to estimate MFDR from the acceleration signal; however, further analysis using normative speaker populations and individuals with varying voice disorder severity is required.

In order to make the most use of our data without re-using any training data in the test set, we trained 51 separate L1-regularized logistic regression LASSO models. For a fair comparison of the collective performance of these models on test input, we used a uniform threshold of 0.5 to classify the output of each 5-min window passed through the LASSO model. This created a set of predicted binary labels (0, 1) for all windows in any subject’s entire record. The proportion of each subject’s windows that are classified as a 1 in this process is plotted in Figure 8, ranging from 0 to 100%. For example, a subject very near the top of the graph would have had almost all of their 5-min windows over the course of the week classified as a 1. Using this output, we can perform inter-model comparisons. In the paper, we report the “optimal threshold” (0.68) that created the highest accuracy measure. It is possible to improve the sensitivity or specificity of our results by lowering or raising this threshold appropriately.

One of the most challenging aspects of voice treatment is achieving carryover (long-term retention) of newly established vocal behaviors from the clinical setting into the patient’s daily environment (Ziegler et al., 2014). Adding biofeedback capabilities to an ambulatory monitor has significant potential to address this carryover challenge by providing individuals with timely information about their vocal behavior throughout their typical activities of daily living. Pilot work has shown that speakers with normal voices exhibit a biofeedback effect by modifying their SPL levels in response to cueing from an ambulatory voice monitoring device (Van Stan et al., 2015a). Long-term retention, however, was not observed and may require the use of alternative biofeedback schedules (e.g., decreasing the frequency and delaying the presentation of biofeedback) that have been well-studied in the motor learning literature.

## Conclusion

Wearable voice monitoring systems have the potential to provide more reliable and objective measures of voice use that can enhance the diagnostic and treatment strategies for common voice disorders. This report provided an overview of our group’s approach to the multilateral characterization and classification of common types of voice disorders using a smartphone-based ambulatory voice health monitor. Preliminary results illustrate the potential for the three analysis approaches studied to help improve assessment and treatment for hyperfunctional voice disorders. Delineating detrimental vocal behaviors may aid in providing real-time biofeedback to a speaker to facilitate the adoption of healthier voice production into everyday use.

## Conflict of Interest Statement

Patent application for methodology of subglottal impedance-based inverse filtering: Matías Zañartu, Ho J. C., Daryush D. Mehta, Wodicka G. R., Robert E. Hillman. System and methods for evaluating vocal function using an impedance-based inverse filtering of neck surface acceleration. International Patent Publication Number WO 2012/112985. Published August 23, 2012. The Guest Associate Editor, Athanasios Tsanas, declares that despite having collaborated with the author, Matías Zañartu, the review process was handled objectively and no conflict of interest exists. The authors declare that the research was conducted in the absence of any commercial or financial relationships that could be construed as a potential conflict of interest.
